# The step in time study: A feasibility study of a mobile app for measuring walking ability after massage treatment in patients with osteoarthritis

**DOI:** 10.1186/s12906-023-03898-w

**Published:** 2023-03-30

**Authors:** Sandra Grace, Roger Engel, Larisa Ariadne Justine Barnes, Joanne Bradbury

**Affiliations:** 1 Faculty of Health, Southern Cross University, Lismore, NSW Australia; 2 Faculty of Medicine, Health and Human Sciences, Macquarie University, Sydney, NSW Australia; 3 Faculty of Medicine and Health, University Center for Rural Health, The University of Sydney, Lismore, NSW Australia

**Keywords:** Feasibility study, Osteoarthritis, Massage therapy, Limitation, mobility, Walking speed, Mobile applications

## Abstract

**Background:**

Massage therapy is a popular intervention for those suffering osteoarthritis, however, there is a paucity of evidence to support its effectiveness in osteoarthritis. A simple measure that could potentially assess the benefits of massage treatment is walking speed which is a predictor of mobility and survival length, particularly in ageing populations. The primary aim of the study was to assess the feasibility of using a phone app to measure walking ability in people with osteoarthritis.

**Methods:**

This feasibility study used a prospective, observational design to collect data from massage practitioners and their clients over a 5-week period. Feasibility outcomes included practitioner and client recruitment and protocol compliance. The app *MapMyWalk* was used to record average speed for each walk. Pre-study surveys and post-study focus groups were conducted. Clients received massage therapy in a massage clinic and were instructed to walk in their own local community for 10 min every other day. Focus group data were analysed thematically. Qualitative data from clients’ pain and mobility diaries were reported descriptively. Average walking speeds were graphed for each participant in relation to massage treatments.

**Results:**

Fifty-three practitioners expressed interest in the study, 13 completed the training, with 11 successfully recruiting 26 clients, 22 of whom completed the study. 90% of practitioners collected all required data. A strong motivation for participating practitioners was to contribute to evidence for massage therapy. Client compliance with using the app was high, but low for completing pain and mobility diaries. Average speed remained unchanged for 15 (68%) clients and decreased for seven (32%). Maximum speed increased for 11 (50%) clients, decreased for nine (41%) and remained unchanged for two (9%). However, data retrieved from the app were unreliable for walking speed.

**Conclusions:**

This study demonstrated that it is feasible to recruit massage practitioners and their clients for a study involving mobile/wearable technology to measure changes in walking speed following massage therapy. The results support the development of a larger randomised clinical trial using purpose-built mobile/wearable technology to measure the medium and long-term effects of massage therapy on people with osteoarthritis.

**Supplementary Information:**

The online version contains supplementary material available at 10.1186/s12906-023-03898-w.

## Background

### Digital health

The challenges for health systems associated with ageing populations and the increasing prevalence of chronic diseases have driven explorations for ways to provide safe, high quality, equitable and sustainable health care using digital health technologies [1]. Digital health platforms, such as telehealth technology, wearable health gadgets, and consumer-facing mobile apps, may serve as useful contributors to both healthcare and research outcomes [2]. For example, digital health technologies have been associated with increased client self-management, compliance, improved education and monitoring [3–5]. These benefits are forecasted to significantly improve health outcomes for those with chronic conditions, given the unprecedented access to remote clients.

The health and fitness wearables market has undergone significant growth [6, 7], which presents new opportunities to efficiently collect and monitor client data (in person or remotely) to improve healthcare consumers’ outcomes, including facilitating accurate and timely diagnosis and intervention [8] and the quality of the care they receive [2]. Challenges highlighted by the Australian Institute of Health and Welfare [8] (e.g. equal access, continuous and accurate transfer of information between systems, ensuring everyone understands the ownership and use of personal data, and protecting sensitive information) need to be negotiated in all instances of their use.

### Massage therapy and chronic health conditions

Studies over many years have confirmed the uptake of natural medicine products and services for the treatment and management of chronic health conditions [9–11]. Massage therapy has been particularly popular for those suffering osteoarthritis [9, 12–14], however evidence supporting massage therapy intervention for osteoarthritis has been hampered by the low/moderate quality of the studies, including high risk of bias [14, 15]. The few systematic reviews and clinical practice guidelines that have been published suggest that massage therapy may lead to short term improvements in pain, stiffness and functional scores in those with knee and hip osteoarthritis [14, 16]. However, given the uptake of massage therapy among people with osteoarthritis, further research is warranted. A mobile or wearable app with the capacity to collect large volumes of data quickly [17] could have applications in this field. Data could be analysed and converted into evidence to support massage therapy interventions for osteoarthritis and other chronic health conditions. It could also be used to capture data from individuals in their natural environments, creating opportunities for understanding and individualising treatment approaches.

### Walking speed apps

Walking speed is a simple measure that could be used to assess the benefits of massage treatment. It is a predictor of mobility and survival length, particularly in an ageing population [18]. Liu et al. [18] reported an 89% increase in all-cause mortality (RR: 1.89, 95%CI: 1.46, 2.46) for the slowest walkers (compared with fastest) in populations of people 65 years and over. A number of government initiatives have emerged to promote active lifestyles, particularly in ageing populations [19, 20]. Apps that can be downloaded onto smartphones to facilitate exercise are promoted on government websites[21]. MapMyWalk (https://www.mapmywalk.com/) is one such app that enables users to track the duration of a walk, distance walked, speed and calories burned during the walk through their mobile smart phones.

Few studies have investigated the effect of massage therapy on walking ability in people with arthritis. Some studies have investigated its use in conjunction with physical exercise for people with other chronic health conditions. For example, a scoping review of the effects of massage therapy on patients with chronic obstructive pulmonary disease found insufficient evidence to support the use of massage therapy alone, however, the authors noted that massage therapy is often proposed as a therapeutic option when used in association with physical exercise with the effect of massage therapy on walking ability assessed by the 6-minute walk test [22]. A randomised controlled trial of 31 people aged 50–85 years found that 30-minute massage therapy, as needed to manage acute flare ups, improved walking ability, also used the 6-minute walk test as the primary outcome, but that 30-minute massage therapy coupled with exercise showed greater improvements [23]. As pain is associated with limiting both walking speed and duration in people with lower limb osteoarthritis [24, 25], massage therapy has the potential to increase walking speed through a reduction in pain in the affected joint. Further investigations of the impact of massage therapy on walking ability could therefore improve our understanding of its contribution to reducing all-cause mortality.

## Methods

### Aims

The primary aim of the study was to assess the feasibility of using a phone app to measure walking ability in people with osteoarthritis. The secondary aim was to determine whether the app could detect a change in walking ability following massage therapy intervention.

### Study design

This feasibility study used a mixed-methods prospective, observational study design to collect data from both clients and massage practitioners over a 4 to 6-week period. Feasibility outcomes included practitioner and client recruitment and protocol compliance. Pre-study surveys and focus group discussions where practitioners were able to share their experiences of and confidence in participating in the research added further insights to the feasibility of this type of study among massage practitioners.

### Settings

Massage practitioners were situated in their own practices, which were located in metropolitan and rural regions in Queensland, New South Wales, South Australia, Victoria and Western Australia. Clients received massage therapy in their usual massage practitioners’ clinics and walked in their local communities.

### Recruitment of massage practitioners and clients

Recruitment of massage practitioners occurred between March and April 2021 and clients between June and November 2021. Massage practitioners were invited to participate via a nation-wide call promoting the study in the Journal of the Australian Traditional Medicine Society (ATMS) and the ATMS subscription electronic digital messaging service. Recruitment was extended to other massage therapy professional associations who used similar strategies to notify their members. Additionally, a research assistant contacted massage practitioners by telephone and email to invite them to the study, using directories from professional massage associations. Interested massage practitioners were invited to attend a 2-hour on-line training session to learn more about the study, their role in the research and the availability of an honorarium for participating. Following the training session, massage practitioners who consented to join the study were then asked to assist with recruiting two or more clients to participate in the study by displaying a flyer in their clinics that referred them to a study research assistant. Massage practitioners were also instructed how to complete a client report form after every massage treatment provided as part of the study.

Interested clients contacted the research assistant by telephone and email. The research assistant then explained the study, detailed eligibility for participation, discussed what participation entailed, and answered any questions the clients had. Clients had the option of completing an online consent form or emailing the signed consent form to the research assistant. Eligibility criteria for massage therapy practitioners and massage clients are detailed in Table 1.

**Table 1:** Eligibility criteria for practitioners and clients

Eligibility criteria – massage practitioners • Accredited massage therapy practitioner (member of an Australian massage or complementary medicine professional association) • Willing to assist with recruiting at least two clients with chronic osteoarthritis to the study • Willing to provide informed consent and to comply with the study protocols
**Eligibility criteria – massage clients** • Over 18 years of age • Diagnosis of osteoarthritis in any joint by a GP or medical specialist • Willing to provide informed consent and comply with the study protocols

### Training sessions for massage practitioners

The training sessions were delivered online via Zoom (https://zoom.us/) to facilitate nation-wide participation. Sessions commenced with an overview of the study and its significance. This was followed by a presentation from the research assistant responsible for managing data collection from practitioners (via Client Report Forms, pre- and post-study questionnaires, focus group discussions), and from the research assistant responsible for managing data collection from clients (walking data from the app, pain and mobility diaries). Finally, the app was demonstrated to practitioners, who were also encouraged to practise using the app before the study so that they could advise and assist their clients.

### The app

MapMyWalk (https://www.mapmywalk.com/) is a popular mobile app that is compatible with both Apple and Android mobile phones. The app is simple to use and provides users with data on average speed, distance walked, start time, and walk duration [26]. However, reliability and validity of the app have not yet been established for all populations.

### Data collection

Data collection from massage practitioners and clients occurred continuously from June through December 2021.

### Massage practitioner data

Participating massage practitioners completed a short online pre-study questionnaire about previous research experience, their confidence in participating in the study and their expected outcomes. They were also invited to attend a one-hour focus group discussion at the end of the data collection period. Focus group discussions were held for practitioners in which they discussed their experiences and perceptions of the study, including suggestions for improving the study and reflections on their learnings about research. Focus group discussions were held virtually using the zoom platform. Recordings were transcribed and checked against the recordings for accuracy before analysis.

### Client data

Baseline data were collected at enrolment including age, gender, height, weight, joint(s) affected by osteoarthritis, length of time since osteoarthritis diagnosis, and current medications. Clients were required to undertake between one and four massage treatments over a 4-week period, as determined by their normal clinical management. Clients were instructed to choose a walk they could comfortably complete every other day, or a minimum of three times a week over a 5-week period.

Clients were asked to walk with their phone over a 5-week period, at the same time each day, and along the same route if possible. To collect walking speed data, clients were instructed to: (i) download the app to their mobile phone; (ii) enable the app and walk for a minimum of 10 min; (iii) take a screen shot of the results at the end of each walk and text or email it to the research assistant; and (iv) turn off the mobile app between walks. A research assistant provided technical assistance with these processes by telephone and email as needed. Clients were also asked to keep a diary of unusual events (e.g. holidays, changes in exercise routine, illness) and a weekly summary of subjective perceptions of pain and mobility using an online or paper diary. Clients self-assessed their pain levels using a 0–10 visual analogue scale (VAS) [27] (0 = no pain at all; 10 = the worst pain you can imagine). Regarding mobility, clients were asked to provide free text answers to the question: *How was your mobility this week?*

### Outcomes

The primary outcome was the feasibility of using a phone app to assess walking speed following massage therapy intervention for clients with osteoarthritis. The secondary outcome was the feasibility of the app to detect changes in average and maximum walking speed. Details of feasibility outcomes are outlined in Table 2.

**Table 2:** Feasibility outcomes and data sources

Feasibility Outcomes		Data source
*Feasibility outcomes - massage practitioners*		
i) Feasibility of recruiting massage practitioners for practice-based research	• Number of expressions of interest received • Number of practitioners who enrolled and completed the training • Time to recruit practitioners • Number of practitioners who completed the study	• Research logs
ii) Feasibility of massage practitioners recruiting their clients for practice-based clinical research	• Number of clients enrolled • Number of clients who completed the study • Time to recruit clients	• Research logs
iii) Practitioners’ compliance with study protocols	• Number of practitioners who submitted all client report forms	• Client report forms submitted to research assistant
iv) Practitioners’ knowledge of and confidence in research pre-study	• Previous involvement in research (qualitative responses) • Confidence in managing the research in their clinics (qualitative responses and a sliding scale 0-100, 0 = no confidence; 100 = complete confidence) • Confidence that the study would be able to measure effectiveness of massage treatment (qualitative responses)	• Online pre-study survey
v) Practitioners’ post-study reflections on participating in the study	• Focus group discussions used a semi-structured interview guide to explore practitioners’ experiences of participation	• Focus group discussions post-study
***Feasibility outcomes - clients***		
vi) Client compliance with using the app	• Ease of teaching clients to use the app • Number of clients who submitted all walk data	• Research logs
vii) Client subjective perceptions of pain and mobility (measured weekly by self-report in pain and mobility diaries)	• Pain - measured by a 0–10 visual analogue scale [27] (0 = no pain at all; 10 = the worst pain you can imagine) • Mobility - self-reported qualitative data	• Client pain and mobility diaries
viii) Client subjective perceptions of pain and mobility (as recorded by practitioners in Client Report Forms)	• Pain- measured by a 0–10 visual analogue scale [27] (0 = no pain at all; 10 = the worst pain you can imagine) • Mobility - self-reported qualitative data	• Client Report Forms completed by practitioners at the beginning of each client visit as part of medical history
***Feasibility outcomes – phone app measures***		
Feasibility of phone app measures to detect actual changes in average and maximum walking speed	• Changes in average walking speed over 5-weeks as measured by the app • Changes in maximum walking speed over 5-weeks as measured by the app	• Data from the app recording average and maximum walking speeds for each walk taken by a client

### Study size

The study aimed to recruit a convenience sample of 30 massage practitioners and 100 clients (that is, approximately 3 clients per practitioner). As this was a feasibility study, the sample size was informed by the nature of the feasibility factors under consideration, including duration and ease of recruitment for both practitioners and patients, and the types of data to be collected and analysed.

### Data analysis

#### Qualitative data analyses

Before data analysis commenced and throughout the analysis, the two members of the research team conducting the qualitative data analysis (SG and LB) reflected on the influence their own backgrounds and beliefs might have had on their interpretive analyses. SG and LB acknowledged that their complementary medicine backgrounds in practice, education and research may influence relationships with study participants, data interpretation and analyses. As LB communicated regularly with trial participants during recruitment and data collection, she did not facilitate the focus groups to prevent the possibility that her presence could influence participants’ discussion and participation. While SG and LB’s complementary medicine backgrounds could potentially influence their interpretations of data, they also acknowledged that their backgrounds could potentially increase participants’ trust and willingness to participate in the research and add to the richness of their engagement with the data.

Qualitative data from the focus group discussions with massage practitioners were analysed using thematic analysis as described by Braun and Clarke [28, 29]. During analysis, SG and LB independently analysed transcripts by hand independently after reading them numerous times to ensure a thorough understanding of the data. Themes were grouped together for analysis. All transcripts were coded by SG and LB separately before SG and LB met to discuss and identify and refine themes for final analysis, ensuring codes were applied consistently and achieving richer interpretations of meaning [30].

All client data were de-identified before analyses. Qualitative data from clients’ pain and mobility diaries was grouped and reported descriptively. Their analyses represent SG and LB’s “reflective and thoughtful engagement” with the data and the analytic process [29], ^p594^.

### Statistical methods

Individual trajectories of walking speed over time were plotted in relation to massage treatment times. Graphs were visually inspected for patterns around the massage treatment times and for overall trends over time: increasing, decreasing or remaining unchanged. Descriptive statistics were used to summarise the data for average and maximum walking speed. In addition, the number and percentage of clients whose walking speed were either increased, decreased or remained the same were calculated.

## Results

### Recruitment and participation

**Fig. 1:**
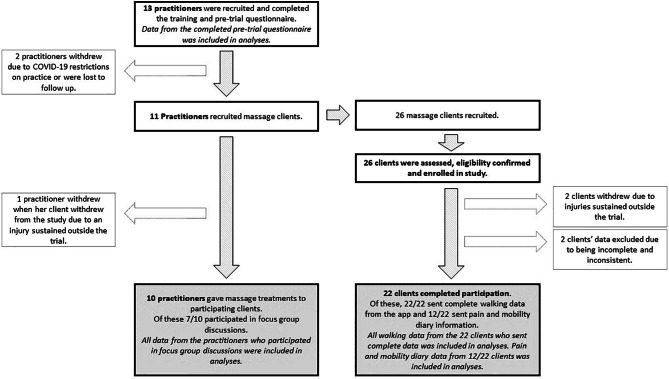
Results of recruitment and participation in the study

Thirteen massage practitioners completed the training and were recruited to the study with eleven going on to successfully recruit clients to the study and 10 completing the study. The 11 massage practitioners recruited 26 massage clients who were assessed for eligibility and included in the study. Of these, 22 completed the study. Results of recruitment and participation are illustrated in Fig. 1.

### Massage practitioners

Eleven massage practitioners were female and one was male. The massage practitioners practised in New South Wales (n = 5), Queensland (n = 3), Western Australia (n = 2), South Australia (n = 1) and Victoria (n = 1)). Eight practised in metropolitan locations and four in rural areas.

### Massage clients

Twenty-two clients completed the study, 16 (73%) were female and 6 (27%) were male. The minimum age of clients was 44 years and the maximum 85 years, with a mean age of 64.3 (SD = 10.6) years. The mean weight at baseline was 83 (SD = 15.4) kg. The mean BMI of clients was 29.9 (SD = 4.5). Most of the clients (n = 12, 55%) reported their primary location of osteoarthritis as the knee, with osteoarthritis of the spine being the next most reported site (n = 6 clients, 27%) (Table 3). Regarding prior confidence in using an app, 14 (64)% rated themselves as reasonably confident, and 8 (36%) felt very confident in using an app.

**Table 3:** Primary location of clients’ osteoarthritic pain

Primary location of osteoarthritic pain	Frequency (n)	Percent (%)
Ankle/foot	1	5
Hip	2	9
Knee	12	55
Shoulder	1	5
Spine	6	27
Total	25	100

### Feasibility outcomes – massage practitioners

#### Feasibility of recruiting massage practitioners for practice-based research

The recruitment period spanned 2 months (March-April 2021). A total of 53 massage practitioners responded to a call for Expressions of Interest in the study. Each was sent a copy of the Participant Information Sheet and Consent From and a follow-up email. Of those 13 were trained and 11 went on to enrol. The main reasons cited for not enrolling were high client volume and potential loss of income associated with voluntary participation in the study. One practitioner was unable to recruit clients in the time frame required for inclusion in the study; 10 practitioners completed the study.

#### Feasibility of massage practitioners recruiting their clients for practice-based clinical research

Twenty-six clients were enrolled in the study over a five month period (June - November 2021). Of these, 22 fully participated and completed the study, two withdrew from the study after suffering injuries unrelated the study, and two clients completed the study but sent incomplete data. Due to COVID-19 restrictions on face-to-face contact with clients, the length of the recruitment period was extended beyond the original projected two-month period. In addition, some clients ‘paused’ their contact with the study during COVID-19 lockdowns, returning once they were able to see their massage practitioner again. The two clients who sent incomplete data were not affected by this ‘pausing’ of participation.

#### Practitioner compliance with study protocol

Nine of the ten practitioners whose clients finished the study completed Client Report Forms for every client visit. One practitioner sent in Client Report Forms with inconsistent visit dates.

#### Practitioners’ knowledge of and confidence in research pre-study

Twelve practitioners completed the pre-study online survey. Of those, nine had not been involved in research previously. Two had experience as participants and one had conducted a systematic review as part of their undergraduate degree.

The majority of massage practitioners felt confident that they could manage the research in their clinics: ten (83%) responded ‘definitely’ and two (17%) responded ‘probably’. Their concerns were about client participation. For example, one participant commented:

Treatment [will be] easily managed in my clinic. Hoping clients are well motivated and have the time to complete. (Practitioner participant #2)

Practitioners were also asked to rate their confidence on a sliding scale from 0 to 100 with 0 meaning low confidence and 100 high. Responses ranged from 59 to 100, with a mean of 85.7 and standard deviation of 14.0.

Practitioners were asked whether they thought the study would be able to measure the effectiveness of their treatment: three (25%) said definitely yes, seven (58%) said probably yes, one (8%) said ‘might or might not’ and one (8%) said ‘probably not’. One practitioner commented on the impact of the pandemic on her clients:

COVID will also differentiate [my clients] as one is an essential worker with a medical background who has not been particularly impacted by COVID - a motivated exerciser who finds any opportunity for activity, whereas the other is an older lady who has felt particularly locked down and isolated through the pandemic. (Practitioner participant #6)

#### Practitioners’ post-study reflections on participating in the study

In the three focus group discussions, practitioners discussed their motivation for joining the study as well as their beliefs about the benefits of massage. Many spoke of a desire to educate the public about these benefits. Practitioners also discussed their beliefs that massage therapy could provide benefits beyond giving clients ‘a luxury or a treat’ such as preventing and treating injuries.

There are still a lot of people who don’t realise how beneficial massage is … it’s a great tool to treat injuries and prevent future injuries. I’m also getting people in when they don’t necessarily have anything wrong with them as such, because prevention is better than cure. (Practitioner participant #1)

Another motivation was to find evidence for massage therapy as an intervention that could reduce pain associated with osteoarthritis.

There’s such a delay in surgery, these days, because they don’t want to do surgery until patients are 60–65 years of age so there are a lot of people in a lot of pain after the age of 40, some even earlier than that. I think if we can find anything that helps [their pain] before they have to go as far as surgery, well that’s a good thing. (Practitioner participant #2)

All practitioners expressed that their experiences of being in the study were enjoyable. They felt they were contributing to something important for their profession.

It’s great to feel like you are part of a team doing something like this. I definitely learned a lot because I’ve never done anything like this before and I was interested to see if I would enjoy it and to have a look to see if it is feasible to use an app to track how clients are doing, so I did really enjoy it … I found it really interesting so far and I think I’m pretty interested in research now so that’s good - something I’ve learnt. (Practitioner participant #3)

The study was conducted during the COVID-19 pandemic and some practitioners were forced to close their practices during government restriction periods. This caused recruitment delays for some, and interruptions between clinic visits for others.

My ladies were actually quite keen and I think they had spoken with [research assistant] and I think [research assistant] had talked to them about the app as well, so when we had to stop and then start up again they were really ready to go and, unfortunately, when it all started well there was just a little bit of having to get back to learning how to use the app again. In dealing with [research assistant] and texting her or myself with any questions, they found it okay. (Practitioner participant #2)

Practitioners commented on how easy participating in the study was, and they put this down to two things: (1) what they were required to do was similar to their usual clinical practice; and (2) they had very good support from the research assistants.

I didn’t sit down for hours afterwards. I just wrote up my notes like I normally would. So for me, it was just part of my day. It just happened to be a study that I was partaking in at the same time. (Practitioner participant #1)

… every aspect of the study had someone that’s sort of in charge of it, and I think that’s [what] helped it flow along quite well in that … I found, as the person doing the massage, it was wonderful when people did have queries regarding the app, I could say I’ll check that with [research assistant]. And also just me having [research assistant] I could go to with any questions I might have had. So it was good in that the support was there, so it made it a good thing to participate in and easy as well. (Practitioner participant #4)

Because of the similarity with their daily practice, some practitioners felt what they learned about research was limited. However, many reported learning a lot about how research works. As one said:

… the methods, the theories and the discussions. It’ll be very interesting to read the outcome of your research and it is a good sort of a way to learn how research works. It is especially difficult in the field of massage therapy, so you chose this app very carefully and smartly so it’s a wonderful way to measure … I learned a lot. (Practitioner participant #5)

An unexpected benefit was that the study was an important motivator for some clients during the lockdown period. They were committed to the study and to their practitioner and so forced themselves to continue walking, even when they felt disinclined. Some clients reported to their practitioner that they became aware of the benefits of walking and continued their walking routine after the study completed.

… he was walking every day on top of that … obviously this trial also helped to motivate him. (Practitioner participant #5)

She’s an older lady who lives by herself. She had a bit of a hiccup in the middle, because she struggled with going back out after COVID. She struggled … she was thinking I’m not sure if I can do it, but she did. She got back out there, and she did it, so I think that kind of forced her through a funny time so that was really helpful. (Practitioner participant #2)

#### Clients’ compliance with using the app

The research assistant reported that it was relatively easy to teach clients how to use the app. She spent between 15 and 60 min with each client teaching them how to use the app. A step-by-step instruction sheet was developed which helped with the training, and the research assistant also provided additional training over the telephone and by email with additional screenshots when necessary. Once the initial training occurred, clients were able to self-manage use of the app and the support required from the research assistant was minimal. Five clients needed text, telephone or email reminders to send data to the research assistant, but most clients did this independently and frequently. For those needing reminding, additional support and training in using the app was offered.

Client compliance with study protocol for sending walk data from the app was relatively high, as 22 sent complete data regularly throughout their participation. Only two clients sent incomplete data (their incomplete data not was included in the analyses).

#### Clients’ subjective perceptions of pain and mobility (self-reported diary data)

**Fig. 2:**
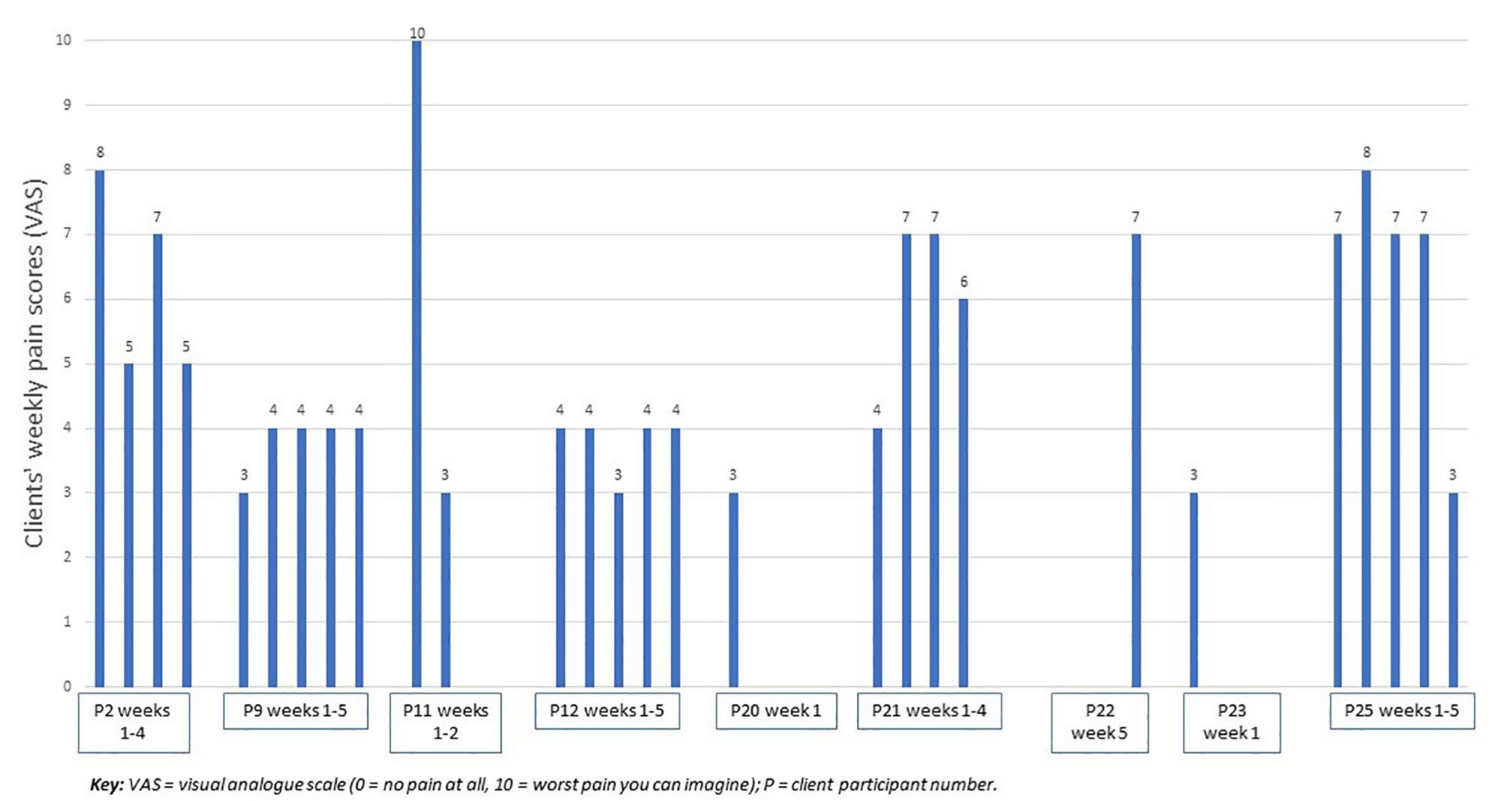
Clients’ weekly pain scores as recorded on a 0–10 visual analogue scale

Compliance with completing pain and mobility diaries was much lower with only 9/22 clients completing any diary data and only four completing the diary for all 5 weeks of participation (Fig. 2). Pain scores ranged from 3 to 10 on the patient reported VAS for Week 1 of the study and from 3 to 7 on the VAS for Week 5 of the study. Some clients’ pain levels stayed relatively stable (e.g., P9 and P12) while other clients reported higher fluctuations in pain levels (e.g. P11). Two clients (P9 and P21) reported worse pain at the end of the study while three clients’ pain decreased (P2, P11, P25) (Fig. 2).

Mobility data reported in clients’ pain and mobility diaries included simple qualitative responses like ‘good’ or ‘poor.’ Some clients included information they felt may have affected their mobility such as:

Mobility wasn’t too bad, having soreness with my left leg shin. (Client #12, Week 2)

Mobility has been good. Bit more stiffer than other weeks and Saturday was awful - I ended up taking 6 anti-inflammatories over the day so I could move. (Client participant #9, Week 4)

When I eventually went for a walk, my legs were very stiff. Knees also painful after the walk. Second walk not as bad. (Client participant #21, Week 2)

When reporting their mobility, some clients took the opportunity to reflect on how they felt the massage therapy was affecting their walking ability.

After a massage I am able to move my back and legs better but after a few days it goes back to niggling and aching pain. (Client participant #9, Week 2)

#### Client subjective perceptions of pain and mobility, as recorded in client report forms

Practitioners collected pain data on a VAS scale of 0–10 (0 = no pain at all; 10 = the worst pain you can imagine) at each clinic visit for all 24 clients. Of these, data for two clients were excluded due to inconsistencies in dates of clinic visits. Of the 22 clients with usable data, visual inspection of pain scores suggested that over the duration of the study pain decreased for eight clients, did not change for nine clients, and increased for five clients (Supplementary File 1).

### Feasibility of using a phone app to assess walking speed following massage therapy intervention for clients with osteoarthritis

#### Changes in average and maximum speed

Twenty-two client participants provided data from their walks over the course of 5 weeks. Average speed remained unchanged for 15 (68%) participants and decreased for 7 (32%) participants. Maximum speed increased for 11 (50%) participants, decreased for 9 (41%) and remained unchanged for 2 (9%) (Table 4). However, on closer inspection, spurious values that could not be accounted for were reported by the app. These resulted in issues of reliability using the maximum walking speed as measured by the app, so there were no further analysis using maximum walking speed.

**Table 4:** Changes in average and maximum walking speed after 5 weeks of the intervention (n = 22 client participants)

	Increase (n, %)	Decrease (n, %)	No change (n, %)
Average speed	0 (0)	7 (32)	15 (68)
Maximum speed	11 (50)	9 (41)	2 (9)

**Fig. 3:**
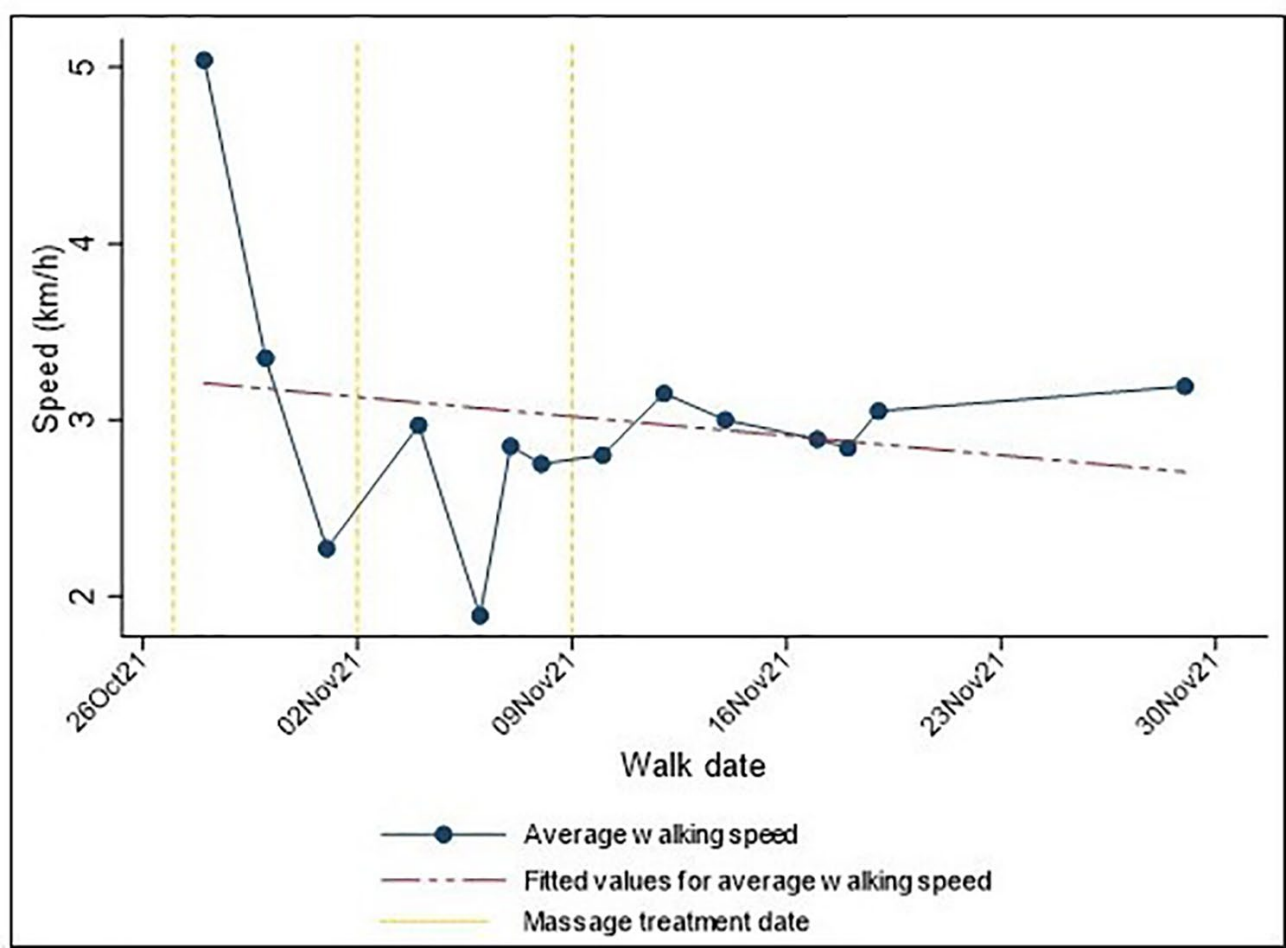
Average walking speed over 5-weeks’ participation in the study for one participant (Client participant #6)

An example of data collected from individuals is illustrated in Fig. 3. For this participant (Client participant #6), average walking speed decreased slightly over time. However, after the first two treatments, there appears to be an improvement in walking speed, as indicated by increased speeds in the walks directly following massage treatment. The response to the third treatment seems to have been delayed, with the second walk following treatment reflecting increased speed. The participant continued to track and send in walking data but did not return for a fourth massage treatment. Graphing individual trajectories over time enabled individual-level analysis of responses to treatment.

## Discussion

### Feasibility

The results of this study suggest that it is feasible to recruit massage therapy practitioners and their clients for an effectiveness trial involving mobile/wearable technology to measure changes in walking speed following massage therapy. Notwithstanding restrictions imposed as a result of the COVID-19 pandemic, practitioners were able to recruit clients with osteoarthritis in a timely manner, in part because massage therapy is seen by the general public as beneficial for osteoarthritis [14]. Compliance with study protocols for using the app (clients) and providing some pain and mobility data (clients and practitioners) was sufficient to determine their feasibility. Having a dedicated research assistant for clients, particularly those who were unfamiliar with the use of phone apps, aided compliance, and is supported by the literature [4].

As healthcare moves towards personalised medicine, individual patient data will become an increasingly important source of information [31, 32]. The use of mobile/wearable technologies like the one used in this study could be extended to include other types of practitioners for measuring the effectiveness of other complementary medicine interventions on people with different chronic diseases. The significance of the project lies in its potential to develop a standardised outcome measure that demonstrates effectiveness of an intervention. Such an approach could be used to assess treatment effects at both the individual and population level. Furthermore, as walking speed is a prognostic indicator in a number of chronic diseases, the effectiveness of different interventions could be directly compared [33–36]. As each person would have their own baseline walking speed, individual treatment effects could be assessed against individual baselines. The development of a common standardised outcome measure such as a walking speed app, may facilitate aggregation of multiple individual-level results to enable inferences to population means, which could, in turn, be used to inform practice.

While the clients of massage practitioners were committed to the research, there were interruptions beyond the control of clients caused by the mass lockdowns during the first and second waves of the COVID-19 pandemic. Clients could not readily access massage treatments, which caused some unanticipated disruptions to the juxta-positioning of the walking data to the timing of the massage treatments. These disruptions may have impacted the results, for example by reducing the capacity of the data to reliably demonstrate possible changes in walking speed after massage treatment within and across individuals’ data. Nevertheless, the data collected from the app allowed for visual inspection of the individual trajectories over time and for exploration of intra-individual and inter-individual patterns in those who were able to undertake more than one treatment. It is probable that multiple massage treatments are required in future studies to establish a relational pattern with the walking data. In addition, other types of contextual data are required to help to understand and interpret any patterns and interruptions noted in the walking time-series data in relation to the massage treatments.

### Building massage practitioners’ research capacity

Collaborative partnerships between researchers and complementary medicine practitioners have been proposed as an enabling strategy to the conduct of research, in order to build the evidence-base in complementary medicine, including massage therapy [37]. Collaborative partnerships between researchers and practitioners can overcome barriers to participation in research by providing practitioners with resources and appropriate research governance to be involved in research, improve their research literacy and competencies [38]. This study exposed a cohort of massage practitioners to practice-based clinical research. Results showed that massage practitioners were prepared to commit their time and resources to clinical-based research, as they felt the research would benefit their profession by building its evidence-base. By engaging practitioners through online collaborative sessions attended by the entire research team, we attempted to cultivate an atmosphere where practitioners could openly discuss their reasons for joining the study, the various stages of the research, their confidence in how they responded and their expectations about outcomes. Sharing knowledge among members of virtual teams has been found to build trust and collaboration [39]. Feedback from practitioners confirmed that there is potential to create a positive experience around research among massage practitioners and supports the proposal that collaborative partnerships can facilitate evidence-based research [37, 38].

### Facilitating exercise

The data analysis found there was no change in the average walking speed of 68% of participants, while 32% of the participants had decreased average walking speeds. This was an unexpected finding that could in part be explained by the disruptions to scheduling massages that occurred in and around the national lockdowns. However, it may be the case that walking speed is a complex functional activity that requires more long-term interventions before improvements can be detected and demonstrated. It may be that there are better, more standardised measures for walking speed such as the 10-meter walk test [40]. However, the exponential accessibility of wearable technologies and digital apps are worthy of further research as they have the potential to collect a huge amount of live data simultaneously, while encouraging people to exercise in a naturalistic environment.

Another unexpected finding reported in this study was that clients who would not have normally exercised due to the COVID-19 restrictions, did exercise as part of participating in the study. Many expressed that they enjoyed walking, that participation motivated them to walk and ‘get out of the house for exercise’ despite government restrictions or self-imposed social isolation due to a fear of contracting COVID-19. Ironically, while COVID-19 warranted the adoption of contingency plans to address the impact of the pandemic on clinical trials [41], this study was able to continue in its original format.

### Recommendations for future research

The willingness of the clients and the practitioners to contribute towards generating an evidence base for massage therapy signals that future studies would be well supported by the industry. Future research could include a fully-powered randomised controlled trial to measure the effect of massage on walking ability in seniors with osteoarthritis, incorporating multiple massage treatment points over a longer period of time to demonstrate clear patterns for individuals. The development of a purpose-built app tailored to suit the collection of data from massage therapy clients is also recommended. This is important to expediate the flow of real-time data from client to researchers and for researchers to have access to the full data in an interactive format, as distinct from summary statistics at discrete measurement points. The purpose-built app could include features that allow participants to record their self-reported pain and mobility data and dates of massage treatment. This would facilitate more comprehensive data collection and help with accurate data analysis.

### Limitations

There were a number of limitations associated with this study. The first was the effect of the COVID-19 pandemic on recruitment of both practitioners and clients with both target sample sizes not reached. While this may affect the generalisability of results regarding efficacy, this study was designed as a feasibility study to inform future research designs, and therefore was not reliant on efficacy measures [42]. Given the circumstances, the sample sizes achieved supported the feasibility of conducting such a study. The second limitation was the delay for some participants between starting the study and completing it. This too, was the result of the COVID-19 pandemic and the restrictions associated with it. It is unlikely that a larger trial conducted in the future would be subject to the same conditions. We therefore argue that this limitation should be considered a one-off. The third limitation was the nature of the data available on the MapMyWalk app. Some of the measures were cumbersome and not suited to clinical massage therapy research, which hindered our ability to determine whether the app could detect a change in walking ability following massage therapy intervention. This was particularly noted in the maximum speed data, where the app, at times, seemed to record spurious values that could not be accounted for in the contextual data and did not seem plausible, and therefore could not be relied upon in the analysis of individual trajectories over time. The average walking speed was the more feasible measure for this app, but may have not been as sensitive to change after massage treatment as maximum walking speed. Developing a purpose-built app that better suits massage therapy clients could overcome such difficulties.

## Conclusion

This study shows that recruiting massage practitioners and their clients for a study involving mobile/wearable technology to measure improvements in walking speed following massage therapy is indeed feasible. Practitioner recruitment indicated that massage practitioners were prepared to commit their time and resources to clinical-based research. Notwithstanding restrictions resulting from the COVID-19 pandemic, most practitioners were able to recruit clients with osteoarthritis in a timely manner. The results from this study support the call for larger randomised clinical trials to measure the medium and long-term effects of massage therapy on walking speed in people with osteoarthritis.

## Electronic supplementary material

Below is the link to the electronic supplementary material.

Supplementary Material 1

Supplementary Material 2

## Data Availability

The datasets generated and/or analysed during the current study are not publicly available as participants did not consent to the sharing of their data. Additional details relating to other aspects of the data are available from the corresponding author on reasonable request.
