# Supplementary material for: The step in time study: A feasibility study of a mobile app for measuring walking ability after massage treatment in patients with osteoarthritis
Source: BMC Complement Med Ther. 2023 Mar 30;23:95. doi: 10.1186/s12906-023-03898-w (PMC10061376; doi:10.1186/s12906-023-03898-w)
Supplement: Supplementary file 1 — Supplementary Material 1 [file 12906_2023_3898_MOESM1_ESM.pdf]

Supplementary file 1. for The Step in Time Study - Data from Client Report Forms completed by massage practitioner participants.

Table S1. Client Report Forms – Pain changes according to Visual Analogue Scales and clients' reported responses to massage therapy.

Client ID	CRF 1	CRF 2	CRF 3	CRF 4	Direction of change in VAS scores*	Clients' final reported response to massage therapy
1	7	6	7	6	Decreased	'Getting back on track- physically a little sore but moving.'
2	6	6	3	5	Decreased	'Felt good for a couple of days then gardening set off px in R hand and deltoid area.'
3	1	3	9	5	Increased	Low back pain subsided, easier to walk, improved mobility.'
4	6	6			Unchanged	'Felt good up until she used vibrating plate.'
5^	8	2	2	2		
6	10	10	10		Unchanged	'Back is good. Hands are great - nil pain in L hand. Only some days about a 2/10 pain in R hand. Much more flexibility in both thumbs and noticeable reduction in swelling - almost normal.'
7^	6	4				
8	10	10	10		Unchanged	'Improvement in walking and in joint pain since using the app; walking everyday.'
9	7	4	3	5	Decreased	'Felt better. More mobility in joints afterwards and during walking.'
10	5	6-7	4-5	6	Increased	'Felt great. Started to hurt 6/10, walking decreased to 4-5/10.'
11	7	7	4	9	Increased	'R knee [initial presenting complaint] feels better to walk on but L knee started to hurt.'
12	5	4	3		Decreased	'After the massage session, which reduced myofascial tension from the body, her rigidity and pain from the area improved significantly. Immediately afterwards, there was no pain in her body. Calmness, relaxation and positivity were strongly felt post massage.'
13	5	2	3		Decreased	'Pain in R sacro-iliac join, hamstrings, iliotibial band +knee. Pain decreased from 3/10 to 0/10 with increase of functionality.'
14	2	7	5	7	Increased	Not recorded
15	5	4	4	4	Decreased	'Has been feeling benefits from massage and looks forward to her visit, pain relief noticeable.'
16	Client withdrew from study					
17	6	7	8	5-6	Unchanged	'Felt good after massage, helped her a little more than before but pain returned after working and walking.'
18	4	2	3	4	Unchanged	'For a few days had relief.'
19	8	6	3-5	8	Unchanged	'Felt better after each session last week.'
20	7	5	5	4	Decreased	'Pain improves for a few days following. Is enjoying the walking and feet are feeling better.'
21	Client withdrew from study					
22	2	3	4	4	Increased	'Massage helpful as always physically active.'
23	4	4			Unchanged	'The client felt excellent after the last treatment.'
24	4	7	6	4	Unchanged	'They did notice a big improvement with the taping last session.'
25	7.5	6.5	7	7.5	Unchanged	'Felt very mobile after last treatment.'
26	6	3	3		Decreased	Not recorded

**VAS = Visual Analogue Scores (0 = no pain at all; 10 = the worst pain you can imagine) recorded at initial visit compared to VAS recorded at final visit.*

^ Data provided on the Client Report Form was inconsistent with dates reported by clients for massage therapy, so this data was not usable.
