# Supplementary material for: The step in time study: A feasibility study of a mobile app for measuring walking ability after massage treatment in patients with osteoarthritis
Source: BMC Complement Med Ther. 2023 Mar 30;23:95. doi: 10.1186/s12906-023-03898-w (PMC10061376; doi:10.1186/s12906-023-03898-w)
Supplement: Supplementary file 2 — Supplementary Material 2 [file 12906_2023_3898_MOESM2_ESM.pdf]

Supplementary File 2. STROBE Statement for The Step in Time Study.

Table S2. STROBE[1] Statement—checklist of items that should be included in reports of observational studies

	Item No	Recommendation	Section of the paper
**Title and abstract**	1	(*a*) Indicate the study's design with a commonly used term in the title or the abstract	Title
		(*b*) Provide in the abstract an informative and balanced summary of what was done and what was found	Abstract
**Introduction**			
Background/rationale	2	Explain the scientific background and rationale for the investigation being reported	Introduction – Background and rationale
Objectives	3	State specific objectives, including any prespecified hypotheses	Methods, Aims
**Methods**			
Study design	4	Present key elements of study design early in the paper	Methods, Study design
Setting	5	Describe the setting, locations, and relevant dates, including periods of recruitment, exposure, follow-up, and data collection	Methods, Settings
Participants	6	(*a*) *Cohort study*—Give the eligibility criteria, and the sources and methods of selection of participants. Describe methods of follow-up<br>~~*Case-control study*—Give the eligibility criteria, and the sources and methods of case ascertainment and control selection. Give the rationale for the choice of cases and controls~~<br>~~*Cross-sectional study*—Give the eligibility criteria, and the sources and methods of selection of participants~~	Methods, Recruitment of massage therapists and clients<br>Methods, Data collection.
		(*b*) *Cohort study*—For matched studies, give matching criteria and number of exposed and unexposed<br>~~*Case-control study*—For matched studies, give matching criteria and the number of controls per case~~	Not applicable
Variables	7	Clearly define all outcomes, exposures, predictors, potential confounders, and effect modifiers. Give diagnostic criteria, if applicable	Methods, Outcomes, including Table 2
Data sources/ measurement	8*	For each variable of interest, give sources of data and details of methods of assessment (measurement). Describe comparability of assessment methods if there is more than one group	Methods, Data collection
Bias	9	Describe any efforts to address potential sources of bias	Not applicable

Study size	10	Explain how the study size was arrived at<br>*Note – to meet feasibility outcomes, the aim was to recruit a convenience sample size.*	Methods, Study size
Quantitative variables	11	Explain how quantitative variables were handled in the analyses. If applicable, describe which groupings were chosen and why	Methods, Data analysis
Statistical methods	12	(*a*) Describe all statistical methods, including those used to control for confounding	Not applicable
		(*b*) Describe any methods used to examine subgroups and interactions	Not applicable
		(*c*) Explain how missing data were addressed	Not applicable
		(*d*) *Cohort study*—If applicable, explain how loss to follow-up was addressed<br>~~*Case-control study*—If applicable, explain how matching of cases and controls was addressed~~<br>~~*Cross-sectional study*—If applicable, describe analytical methods taking account of sampling strategy~~	Not applicable
		(*e*) Describe any sensitivity analyses	Not applicable
Qualitative data	*12.1*	*Please note - As this study was a mixed methods study, Qualitative data has been added to this checklist as item 12.1*	Methods, Data analysis, Qualitative data analysis
**Results**			
Participants	13*	(a) Report numbers of individuals at each stage of study—e.g., numbers potentially eligible, examined for eligibility, confirmed eligible, included in the study, completing follow-up, and analysed	Results, Recruitment and participation
		(b) Give reasons for non-participation at each stage	Results, Figure 1
		(c) Consider use of a flow diagram	Results, Figure 1
Descriptive data	14*	(a) Give characteristics of study participants (e.g., demographic, clinical, social) and information on exposures and potential confounders	Results, Table 3
		(b) Indicate number of participants with missing data for each variable of interest	Results, Figure 1
		(c) *Cohort study*—Summarise follow-up time (e.g., average and total amount)	Not applicable
Outcome data	15*	*Cohort study*—Report numbers of outcome events or summary measures over time	Results
		~~*Case-control study*—Report numbers in each exposure category, or summary measures of exposure~~	Not applicable
		~~*Cross-sectional study*—Report numbers of outcome events or summary measures~~	Not applicable
Main results	16	(*a*) Give unadjusted estimates and, if applicable, confounder-adjusted estimates and their precision (e.g., 95% confidence interval). Make clear which confounders were adjusted for and why they were included	Not applicable
		(*b*) Report category boundaries when continuous variables were categorized	Not applicable
		(*c*) If relevant, consider translating estimates of relative risk into absolute risk for a meaningful time period	Not applicable
Other analyses	17	Report other analyses done—e.g., analyses of subgroups and interactions, and sensitivity analyses	Not applicable

Qualitative analyses	17.1	*Please note - As this study was a mixed methods study, results of analysis of qualitative data has been added to this checklist as item 17.1.*<br>Results, Feasibility outcomes – massage practitioners, v. Practitioners' post-study reflections on participating in the study and<br>Results, Feasibility outcomes, vii. Clients' subjective perceptions of pain and mobility (self-reported diary data)	Results, Feasibility outcomes v. and vii.
**Discussion**			
Key results	18	Summarise key results with reference to study objectives	Discussion
Limitations	19	Discuss limitations of the study, taking into account sources of potential bias or imprecision. Discuss both direction and magnitude of any potential bias	Discussion, Limitations
Interpretation	20	Give a cautious overall interpretation of results considering objectives, limitations, multiplicity of analyses, results from similar studies, and other relevant evidence	Conclusion
Generalisability	21	Discuss the generalisability (external validity) of the study results	Discussion, Limitations
**Other information**			
Funding	22	Give the source of funding and the role of the funders for the present study and, if applicable, for the original study on which the present article is based	Declarations

N.B. The STROBE statement says to "Give information separately for cases and controls in case-control studies and, if applicable, for exposed and unexposed groups in cohort and cross-sectional studies." *This was not applicable for the study reported here.*

Note: An Explanation and Elaboration article discusses each checklist item and gives methodological background and published examples of transparent reporting. The STROBE checklist is best used in conjunction with this article (freely available on the Web sites of PLoS Medicine at http://www.plosmedicine.org/, Annals of Internal Medicine at http://www.annals.org/, and Epidemiology at http://www.epidem.com/). Information on the STROBE Initiative is available at www.strobe-statement.org.

Reference

1. von Elm E, Altman DG, Egger M, Pocock SJ, Gotzsche PC, Vandenbroucke JP. The Strengthening the Reporting of Observational Studies in Epidemiology (STROBE) Statement: guidelines for reporting observational studies. Ann Intern Med. 2007; 147(8):573-577.
